# Challenges for the legislation enabling egg donation in Switzerland

**DOI:** 10.1177/09685332241269583

**Published:** 2024-09-19

**Authors:** Andrea Martani, Nathalie Neeser, Nicolas Vulliemoz, Guido Pennings

**Affiliations:** Institute for Biomedical Ethics, University of Basel, Switzerland; Centre de Procréation Médicalement Assistée (CPMA), Switzerland; Department of Philosophy and Moral Sciences, Bioethics Institute Ghent, Ghent University, Belgium

**Keywords:** Medically assisted reproduction, health law, egg donation, age limits for assisted reproduction, egg sharing

## Abstract

Switzerland is one of the most restrictive countries in Europe when it comes to the regulation of egg donation in medically assisted reproduction (MAR). Indeed, even after the introduction of modifications to the law regulating reproductive medicine allowing embryo culture, embryo freezing, and preimplantation genetic testing, egg donation has remained completely forbidden. The absolute ban on egg donation is heavily discussed in academia, society, and politics. After many failed attempts, this prohibition is now on its way to be lifted, after agreement was reached in the legislative institutions. The forthcoming legalisation of egg donation raises, however, several questions on how some aspects of this practice will be regulated. In this contribution, we briefly review the reasons why a ban on egg donation has been present for so long in Switzerland, to then analyse two issues raised by the commitment to lift this ban. First, we reflect on the question of whether the new legislation should introduce chronological age limits for access to heterologous MAR. Second, we consider how the practice of egg sharing could be regulated once egg donation is legal.

## Introduction

The regulation of medically assisted reproduction (MAR) is a contested issue due to its ties with highly-sensitive topics, such as the moral status of embryos and their use in medicine and research, the boundaries of parental claims towards a ‘right’ to have children, and the potential limits to accessing such technologies through social health insurance. As a result, whether such regulations are enshrined in hard law governing access to MAR at a general level, or more specific rules on the reimbursement of these medical services, or even ‘soft law’ such as medical guidelines, norms around MAR vary considerably between countries.^1^ Indeed, many countries present a set of restrictive conditions to access (e.g. by imposing age limits for the aspiring parents) or to use (e.g. by limiting the number of reimbursed treatment cycles) such services.^2^ The existence of a certain degree of diversity in how MAR is regulated has been upheld by the European Court of Human Rights (ECHR). Indeed, the latter – albeit with certain limits and not without criticism^3^ – has been defending the right of states to regulate issues related to reproductive healthcare and reproductive rights with a considerable amount of freedom by applying the doctrine of the margin of appreciation.^4^

Despite the aforementioned diversity, there are a few matters where legal rules on MAR have been converging across Europe. One of these concerns the permissibility of egg donation. The European Atlas of fertility treatment policies^5^ shows that some form of egg donation is now allowed almost all over Europe. Even countries that used to prohibit it have been adapting their legislations in the last few years. Austria was the country whose restrictive legislation led to the controversial decision of the ECHR deeming the prohibition of egg donation as legal, because it allegedly fell within the state’s margin of appreciation.^6^ Yet, the country reformed its legislation in 2015 to eventually allow this practice.^7^ The Italian law on MAR in its initial version also only permitted homologous procedures (i.e. using gametes from the aspiring parents), but the Constitutional Court removed this limitation in 2014.^8^ Although many countries have been moving along these lines, a notable exception to the general permissibility of egg donation in Europe is that of Switzerland.^9^ Indeed, art. 4 of the piece of legislation on MAR^10^ completely outlaws – together with surrogate motherhood and embryo donation – the practice of egg donation.

After almost a decade of failed attempts by different political forces to uplift the ban, in 2022, the Swiss Federal parliament paved the way for this to eventually happen. However, while there is now increasing political support for legalising egg donation, doubts remain concerning *how* this practice – once legalised in the coming years – should be regulated. In this contribution, we focus on the latter issue and reflect on two unresolved matters for the upcoming legalisation of egg donation, which we identified in the context of larger study we are conducting on Advanced Parental Age and MAR (more details below). Our objective is twofold: First, we want to analyse two complex issues that need addressing when legalising egg donation in any system of law; second, we want to propose regulatory solutions for the Swiss context, but which can also help inform the debate in other countries. To do so, we start by briefly reviewing how the ban on egg donation came to be initially included in the Swiss law on MAR and the grounds based on which it was defended. We then summarise the political and legal process that has been leading to the decision to lift such ban. In the section titled ‘Two open questions’, we turn to two unresolved questions that the legislator needs to address once the absolute prohibition is eliminated. For this section, we also draw from the experience that we have been accumulating as part of a research project on the phenomenon of advanced parental ageing in the context of MAR. More specifically, we show that one issue which the Swiss legislator needs to consider is whether to introduce chronological age limits for the recipients of egg donation, once the general ban is lifted. Second, we argue that there is a need to determine how the practice of egg sharing will be managed. In the section ‘Propositions for addressing the two legal issues’, we suggest potential solutions for the Swiss context, but also include considerations that are relevant for other legal systems. Finally, in the concluding remarks, we underscore that lifting this ban is a sound and justified choice, but we also recommend an attentive reflection on how egg donation should be regulated.

## The prohibition of egg donation: reviewing the origin of the restrictive Swiss approach

The prohibition of egg donation in Switzerland is nested within a Federal piece of legislation on MAR passed in 1998. Beforehand, MAR was mainly regulated through the ‘Medico-ethical guidelines for medically Assisted Reproduction’ approved by the Swiss Academy of Medical Sciences in 1990^11^ which, interestingly, permitted egg donation. In addition, some Cantons (the Federal states which Switzerland is comprised of) had promulgated specific cantonal laws on this matter, which were – however – challenged before some courts^12^. These were substituted by the Federal law of 1998, which was the product of more than a decade of discussion in the complex Swiss law-making infrastructure. Indeed, the process that lead to a Federal regulation of MAR can be traced back to the submission of a Popular Initiative in 1987.^13^ Popular initiatives are instruments of Swiss direct democracy which allow citizens to submit requests to the Federal institutions to change or add articles to the Constitution, as long as they collect a minimum of 100,000 signatures within 18 months. Popular initiatives are very powerful, since they lead to a country-wide vote on whether they should be implemented or rejected, even if the government and parliament disagree with the content. In this case, a popular initiative titled ‘Against the abuses in the field of reproductive medicine and gene technology in humans’ was submitted to the parliament, calling for the addition of several articles to the Swiss constitution aimed at prohibiting or restricting many procedures related to reproductive medicine. Stimulated by this Popular initiative, the Federal institutions prepared a counter proposal,^14^ which – although being a compromise – picked up much of the restrictive approach demanded by the popular initiative. This counter proposal was then voted by Swiss citizens, thus determining the addition of art.119 to the Swiss constitution as formulated by the counter proposal. This set some basic rules related to reproductive medicine (including, e.g. a constitutional prohibition of surrogacy and the recognition of a right to know one’s genetic origin) and also gave the Federal parliament the power to emanate a specific piece of legislation on MAR. The parliament used this power, and in 1998, the Federal law on MAR – officially named ‘Federal Act on Medically Assisted Reproduction’ or ‘Reproductive Medicine Act’ in its short form^15^ – was passed.

In this law, art. 4 is titled ‘Prohibited practices’ and states that ‘Ovum and embryo donation and surrogate motherhood are prohibited’. Embryo donation and surrogate motherhood are also prohibited directly in newly added art. 119 of the Constitution; therefore, they had to be prohibited also in the Reproductive Medicine Act. On the contrary, egg donation is not mentioned in the Constitution, so the Parliament autonomously decided to add it to the list of prohibited practices in the initial proposal for the legislation – for reasons which are presented below. Such a decision was very heavily debated. In a first reading of the initial legislative proposal in the Council of States,^16^ a slim majority voted to exclude such a prohibition, so that egg donation would be legal. But the second chamber of the Swiss parliament, the National Council,^17^ overturned this decision by a large majority and voted to keep the general ban on egg donation as it was framed in the original legislative proposal. On a second reading of the proposal, the Council of States eventually accepted this decision, and the final version of the legislation officially promulgated in 1998 contained the blanket prohibition on egg donation.^18^

In the communication that the Swiss government publishes to accompany the new legislative process and to justify the wording and content of the different rules, the prohibition of egg donation was justified based on the following grounds.^19^ First, it was claimed that not enough evidence was available at the time concerning the potential consequences for the child in case of MAR with a donated egg. In addition, it was argued that allowing egg donation would lead to the creation of familial relationships that are otherwise impossible through natural reproduction. In fact – the communication claimed – natural conception would always lead to the birth of a child with a genetic link to the mother, whereas permitting egg donation would violate the legal rule that *mater semper certa est*. The fact that sperm donation, on the contrary, was not prohibited was justified based on the fact that

The presence of a separation between genetic father and socio-legal father through sperm donation mirrors, differently from egg donation, a situation that could also occur naturally: experience showed that [. . .] the partner of the birthing person is not necessarily the biological father of the child.^20^

In sum, as commentators have outlined,^21^ the prohibition of egg donation was anchored by the lawmaker mostly in the argument of ‘naturalness’: The ban was portrayed as justified insofar as it does not lead to a situation that would be impossible in nature. In other words, an empirical observation (i.e. the fact that no mother can naturally give birth to a non–genetically-related child) was used to draw a normative conclusion (i.e. having children without a genetic tie to the mother should not be permitted).

## The forthcoming lift of the absolute ban

The general ban on egg donation and the justification that the legislator used to uphold it have been under severe scrutiny since the very start. Indeed, legal experts have rightly observed that such prohibition cannot be justified based on concerns for the health of the mother or the children, given that now ample evidence exists on the safety of MAR with egg donation, and that this is a routine medical treatment in all countries that allow it.^22^ Moreover, the ban creates discrimination towards women, in that their access to MAR is excluded when the cause of infertility is related to their gametes, whereas men can always turn to the legally permitted sperm donation. It has been convincingly stated that such discrimination cannot be justified based on the aforementioned argument of naturalness, since this is not a satisfying reason to treat the separation between genetic and social parenthood that happens with sperm donation differently from the one that would happen with egg donation.^23^ If anything, as Belser and Jungo highlight,^24^ one could argue that mothers who conceive through egg donation have a stronger relation to the child than the socio-legal father in case of sperm donation, since the mothers carry the child to term and thus have a ‘biological’ (albeit not genetic) connection with it. Moreover, there is no public interest to protect by safeguarding the ‘naturalness of reproduction’, else any MAR (or indeed many other medical interventions) should be prohibited.^25^

Such criticisms have also slowly started to resonate in the political arena, and many initiatives have been introduced to try and lift the general ban.^26^ After many failures, it now seems that the time is ripe for the legal change to happen. In fact, in November 2021, the commission for science, education, and culture of the National Council submitted a motion to the Parliament for the legalisation of egg donation.^27^ The motion called for lifting the ban in order to eliminate discrimination between sterile men (who can benefit from sperm donation) and women (who cannot have access to donated eggs), to reduce the need of citizens to go abroad to receive egg donations, and to join the overwhelming majority of European states, which allow this practice. The motion was opposed by the Federal government, which asked both chambers of the parliament to reject it. However, in March 2022, the motion obtained the majority (107 in favour, 57 against, and 16 abstentions)^28^ in the National Council, and in September 2022, it also passed the vote in the Council of States (22 in favour, 20 against, and 0 abstentions).^29^ In consequence, the government is now obliged to prepare the legislative change by making a proposition containing the terms according to which the legalisation of egg donation should proceed.

## Two open questions

As a consequence of the political process outlined above, chances are very high^30^ that in the near future, the general prohibition of egg donation will eventually be lifted and that Switzerland will join the great majority of European countries, which do not have such blanket bans. Nevertheless, the issue remains of how to concretely regulate egg donation, once permitted. Some proposals have already been advanced, and several matters related to the legalisation of egg donation have already been examined in the public and scientific debate. These include, for example, how to regulate the right to know about one’s genetic origins, or whether donation to a known person (e.g. friend, relative) should be allowed, or whether there should be a limit to the number of cycles to which an egg donor can agree. There are – however – two aspects which did not receive adequate attention, but which are crucial when it comes to the regulation of egg donation, especially in the context of Switzerland.

We identified them as part of our research within A-PAGE, a Swiss-Belgian interdisciplinary project on Family Building at Advanced Parental Age, which explores the moral, legal, and social significance of age in reproduction.^31^ In the framework of this project, we also conducted literature and legal analyses of age limits for different types of MAR and conducted interviews with patients who accessed MAR and with healthcare professionals from reproductive clinics. When considering the issue of egg donation as part of the research, two understudied issues related to the upcoming legalisation of this practice came to the fore. First, the question whether the legalisation of egg donation should be accompanied by the introduction of chronological age limits for parents who make use of this technology. Second, the issue of how the legalisation of egg donation should deal with rules and norms around the procurement of eggs. We will analyse both issues in the following sections.

### The question about a chronological age limit

According to published legal analysis,^32^ the greatest majority of European states which explicitly regulate MAR have chronological age limits for aspiring parents (especially mothers, but also fathers) who desire to access these technologies. Age limits can be described as chronological when they contain a specific number of years in their definition, rather than non-numerical references.^33^ This means that national legislations usually set a specific chronological age (e.g. 45 or 50 for the aspiring mother) beyond which (1) either parents are categorically not allowed to access MAR, or (2) their treatment will not be covered as part of basic public health insurance. In this respect, Swiss law stands out, as it is one of the few that do not contain any chronological age limits for parents. Indeed, the only explicit reference to parental age can be found in art. 3 of the Reproductive Medicine Act, which simply says that:

Assisted reproductive techniques may be used only if the well-being of the child is ensured. They may only be used in couples [. . .] who, on the basis of their age and personal circumstances, are likely to be able to care for and bring up the child until it reaches the age of majority.^34^

Therefore, differently from other legislations, Swiss law gives healthcare practitioners no specific chronological yardstick. If they, based on the conditions of the parents who seek access to MAR, believe that the age/conditions of the parents (collectively as a couple) will likely allow them to take care of the child, healthcare professionals can grant access to reproductive technologies. In operational terms, we have learned from interviews conducted with healthcare professionals that this is usually implemented by asking prospective parents to present basic certificates about their state of health once one parent^35^ is around the age of 60. One healthcare professional we interviewed said, for example:

But when a man [in the couple seeking access to MAR] is around 55 or approaching 60, they naturally have to bring a medical certificate that they are healthy, that they are fit and that you can expect a normal life expectancy, so that the child will actually still be of age, ehm, that the father will still be there. But of course you never know, even with young men you don’t know whether a stroke of fate . . . I mean, there’s always that, of course. I mean, that’s, but that’s just the legal limit that we have to observe.

Based also on the practical experience of one of the authors (NV), we know that this practice is widespread. Reproductive clinics, when the prospective father is around 60, normally require some form of medical or psychological consultation, a general assessment of the health, and decide then collegially on whether they believe that the couple as a whole is likely capable of caring for the child until the age of majority (as stipulated by art. 3).

On top of the limits posed by article 3, Swiss legal experts^36^ have defined that the ban on egg donation generates a second ‘implicit’ age limit, in that women – since they *must* use their own eggs – are *de facto* excluded from MAR access when they approach the age of menopause. Elsewhere,^37^ we defined this as a *biological* age limit, because it denies access to MAR for all women who – as their reproductive organs age – are not able to have any more (fertile) eggs of their own. In this way, the law has *de facto* impeded that women beyond their mid-40s are able to access MAR,^38^ given that extraction of own eggs and achievement of live birth at that stage is very unlikely. In fact, most centres have explicit institutional policies whereby MAR is not offered when the woman is 43 or older, and the justification provided is based on the fact that treatment would border medical futility above this age. For example, one clinic in Zürich (the largest town in Switzerland) writes:^39^

While there are no legal restrictions on the age at which a treatment can be carried out, nature sets some limits to the chances of success of fertility treatment. In principle, we therefore offer in vitro fertilisation (IVF) and intracytoplasmic sperm injection (ICSI) until the woman’s 43rd birthday. Only in a few exceptional cases with above-average conditions does it make sense to exceed this limit by a maximum of 1-2 years.

What is important to underscore is that this has facilitated the application of the general conditions set by art. 3. Indeed, the fact that at least one parent (the mother) of a Swiss aspiring couple trying to access MAR will always *de facto* be young (43 years of age is *very* young in terms of life expectancy) makes it easy to ascertain that the couple will likely be able to care for the child until its age of majority.

However, this may change with the legalisation of egg donation. The latter will entail that also couples where *both* aspiring parents are relatively old can seek access to MAR, since the biological age limit for women (i.e. approaching the age where an attempt to conceive with their own eggs would amount to futile treatment) will fall. In this respect, a recent report by the National Ethics Committee (NEC), a governmental advisory board on matters related to bioethics, argued that the legalisation of egg donation opens up two options for the lawmaker.^40^ On the one hand, an explicit chronological age limit for the aspiring mother receiving the egg donation could be implemented, as this is the case in the overwhelming majority of European states. On the other hand, the current rule (art. 3) – which requires healthcare professionals to consider whether the couple can likely care for the child until the age of majority – could be maintained without any further additions. The NEC speaks in favour of the latter option, arguing that an explicit chronological age limit only for women (i.e. the egg receiver) would be discriminatory, and saying that ‘if the risks [determined by the advanced age of the parents] are too high for the potential child, the doctors are anyway obliged to refuse access to ART [assistive reproductive technologies, another name for MAR]’ (our translation). While these arguments are valid, doubts remain as to the concrete functioning of the law if this option is selected.

Indeed, one reason why the current situation (i.e. the flexible rules of art. 3) is relatively easy to operationalise for healthcare professionals even without a legally-set chronological age limit is that *de facto* all treatments where the parents (especially the woman) are particularly old can be rejected on the grounds of medical futility. MAR with own fresh eggs^41^ above the age of 43–45 years would indeed have almost always negligible chances to lead to a live and healthy pregnancy. This is also the reason why currently many clinics have institutionally-imposed chronological age limits, whereby they do not offer treatment beyond the age of 43–45 years for the mother. In fact, there is a general medical consensus that MAR beyond that age with own eggs would almost amount to futile treatment in the greatest majority of cases. In practice, this means that currently (i.e. as long as egg donation is prohibited):

1. If the aspiring mother in the couple trying to access MAR is of the age range 43–45 years or below and the medical teams consider that MAR with own eggs is still viable, the couple will get access even if the aspiring father is much older (e.g. 55–60 years). The latter may have to provide some basic certification of good health, but it will be easy to conclude that the aspiring parents – as a couple – meet the conditions of art. 3, since the mother will always be young enough (from the perspective of life expectancy) to offer a reasonable guanrantee that the child will be cared for until the age of 18.
2. If two parents come, where the aspiring mother is above 43–45 years of age, access to MAR will almost certainly be refused based on considerations of medical futility. This will happen even if the couple as a whole (let us assume the aspiring father is 48) may still meet the conditions of art. 3.

However, the application of the flexible rules of art. 3 without a specific chronological age limit will become much more difficult once egg donation is allowed. Indeed, studies have shown that women can obtain reasonably high pregnancy rates until their late 50s and early 60s when they use donated eggs.^42^ As a consequence, individual clinics will have much fewer uncontroversial reasons to define when an attempt of achieving pregnancy with the help of MAR is *undoubtedly* futile from a purely medical point of view. At this point, they will be left to take a case-by-case decision for each couple based solely on the considerations enshrined in art. 3 about the probability that aspiring parents will be able to care for the child until the age of majority. As illustrated earlier, until now (i.e. with the ban on egg donation), the application of this standard is relatively easy: At least one parent (the aspiring mother) is rarely older than mid-40s, and – as long as the partner is reasonably healthy – it has been easy to judge the couple as likely capable of caring for the child until its adult age. When egg donation is legalised, healthcare professionals may be in a much more difficult situation. Without a specific chronological age limit, will they be able to apply art. 3 easily and in an unbiased manner? Would they, for example, judge an aspiring couple in the same way, where the aspiring mother is relatively older than the aspiring father?

In short, the question about age limits for aspiring parents accessing MAR with egg donation remains an issue that needs particular discussion during the process of legalising of this practice. Needless to say, the difficulty of solving this question should not be used as an argument to keep egg donation prohibited. On the contrary, settling on age limits should be used as an incentive to discuss how to implement a law that ensures non-discrimination and fair access to MAR and that does not put healthcare professionals in the uncomfortable position of having unclear indication as to how they should apply the flexible standards of art. 3.

### Regulating egg procurement and the delicate practice of egg sharing

One of the most difficult issues connected to the legalisation of egg donation is how to regulate the procurement of eggs. Indeed, scarcity of eggs for MAR is a problem that virtually all countries that legalise this practice face.^43^ Although it is impossible to know exactly to what extent scarcity of eggs will also be an issue in Switzerland, there are very good reasons to suppose that Switzerland will make no exception. Indeed, in a recent interview with a researcher specialised on cross-border reproductive practices in Switzerland, she explained that – given the fact that Swiss law forbid compensations for any donation of human cells, tissues, or organs – there will be very few women available to donate their eggs.^44^ The only empirical evidence available on this topic is a small survey from 2014, where a group of researchers investigated whether women in Switzerland would be potentially available to donate their eggs and which reasons would motivate them to do it.^45^ Out of the 172 respondents, 56.4% declared to be potentially willing to donate their eggs, but – as the authors themselves warn – this relatively high percentage must be taken with reservations, given that the sample was not representative and subject to selection bias. Moreover, as other studies analysing similar questions in other countries point out,^46^ even when many participants to such survey studies say they would donate, this never translates in the same percentage *actually* donating.

The question for the legislator is then how to design a regulatory framework that ensures not only that egg donation is permitted, but also that eggs for the procedure can *actually* be retrieved. One of the most discussed options in the ethico-legal literature concerns payment of donors. In terms of policy, the Ethics Committee of the American Society for Reproductive Medicine recommends particular caution with respect to financial compensation for eggs and warns that ‘Compensation should be fair and should not be an undue enticement that negatively impacts a donor’s ability to make an informed decision’.^47^ In Europe, there is a general consensus that eggs should not be treated and traded as commodities since ‘this would amount to the commercialization of reproduction’,^48^ but that some financial compensation can be given to donors.^49^ However, when it comes to translating these principles into policy, things get more complex. In fact, it is difficult to draw the line between forms of compensation that simply recognise the effort and the trouble egg donors go through, and indemnities that leave donors in a significantly better financial position, to the extent that they are morally equivalent to direct payment for eggs.^50^ Empirical research also confirms that there are different levels of public support for compensation towards gamete donors depending on the level of financial incentives provided to egg donors.^51^ Swiss law generally imposes the principle of gratuity in respect to the donation of human cells and tissues. The current rules on sperm donation (which is already allowed) require explicitly that ‘No payment shall be made for sperm donation as such’.^52^ The reports that discussed the legalisation of egg donation also required to respect the principle of gratuity and conceded only that some form of indemnity to cover the troubles of going through the procedure can be granted. Given the emphasis on the principle of gratuity and the restrictive approach towards compensation that Swiss law has in reproductive medicine,^53^ it is to be expected that such indemnities will remain quite low and will have a limited impact on the recruitment of donors when egg donation is allowed. If this is the case, it is unlikely that a few hundred Swiss francs of compensation will be enough to motivate many people to donate their eggs.^54^ Indeed, even for sperm donation – which in Switzerland is currently regulated by the same principles of gratuity^55^ and altruism – the number of people who give their gametes is rather limited.^56^

There are other options for the law to favour the donation of eggs which do not involve stipulating that high compensation can be given, especially because research shows this is not the only motivator why women share their eggs. As expressed in a report commissioned by the Swiss government in 2014,

Efforts [in designing a legislative and policy framework that facilitates the donation of eggs] can be made at three different levels. The first concerns the drivers, or what motivates a donor to donate; the second concerns the modes of recruitment, or how to reach the donor; and the third concerns the donation sources, which can also vary, or where to find oocytes.^57^

For example, in Belgium, the legal and policy framework has catered for a system where donors are normally friends and relatives, with the possibility for cross-donating between different sets of aspiring parents.^58^ This system delegates the aspiring parents to find the donor and relies on the fact that their motivation may help to find a willing woman in the respective social networks. The system may work very efficiently, but it requires social acceptability of the practice of recruiting among friends and family, which seems present in Belgium, but can be absent elsewhere. Although it has been argued that donation from a relative (or friend) may be a potential solution also for Switzerland,^59^ it is unsure whether this practice will be made legal in the context of egg donation. In fact, sperm donation from a known person is considered unlawful,^60^ and unless such rules are changed, it may be inconsistent to allow it only for egg donation.

Another alternative that is quite widely mentioned in the Swiss context as a potential legal scheme that would facilitate the procurement of eggs is *egg sharing*.^61^ Indeed, a renowned MAR physician in Switzerland has been repeatedly expressing the desirability of implementing this practice.^62^ Moreover, both a governmentally commissioned report and an opinion of the NEC^63^ called for the implementation of egg-sharing schemes together with the legalisation of egg donation. Egg sharing is different from ‘standard’ egg donation, where a woman undergoes hormonal treatment, and the mature eggs are extracted and then given directly to a recipient in need.^64^ It is also different from freeze-and-share practices, where women who decide to freeze their eggs for social (e.g. to try and have genetically-related children also at an advanced parental age) or medical (e.g. to preserve fertility after chemotherapy) reasons agree to give some of their unused frozen eggs to other aspiring parents.^65^ Egg sharing, on the contrary, implies that a woman (the egg sharer), who is herself undergoing MAR with own eggs, pre-emptively agrees to give part of them to another person considering MAR and in need of donor eggs (the egg receiver). As summarised by Blyth,^66^ egg sharing is considered particularly useful because it does not require an additional woman to be exposed to hormonal treatment (since the ‘egg sharer’ is anyway undergoing treatment for her own MAR). Moreover, the egg sharer normally obtains a discount on her treatment in exchange for giving up some of her eggs. This has been described as a win-win situation,^67^ where the egg receiver obtains oocytes and the giver obtains discounted treatment. This is one of the reasons why egg sharing is widely used in the United Kingdom, where coverage of MAR through basic health insurance is limited, and becoming egg sharers is a way for women to access MAR at a lower out-of-pocket cost.

According to Swiss legal commentators, once egg donation is legalised, this will also open up the possibility of practicing egg sharing as a way to procure eggs in Switzerland, and it will also permit to compensate egg sharers by giving them a discount on their treatment.^68^ Commentators consider egg sharing as a rather unproblematic practice in the Swiss context, which can then be legalised together with egg donation. In a statement on egg donation published in 2022 by the NEC, they claimed:

According to the NEC, there are no convincing reasons to forbid egg sharing, i.e. the possibility to donate eggs that were obtained during an IVF treatment and are not necessary required anymore [by the women who donates them]. With egg sharing special attention should be given that the IVF treatment of the donor is not compromised by the donation.^69^

The only potential issues related to egg sharing which have been mentioned^70^ as worthy of attention include (1) the case when too few eggs are retrieved, the right of the (potential) egg sharer to use all the eggs for her own treatment must be respected; (2) if the egg sharer accesses MAR at a discounted price in exchange for providing eggs for someone else, the egg sharer should not feel forced to continue treatment just because of the pressure of the financial consequences (e.g. repeal of the discount); and (3) psychological support must be present, in case the egg sharer does not reach pregnancy, but the egg receiver does.

However, there is one crucial point that is not addressed in the discussion about the legalisation of egg sharing. As Blyth and Golding outline,^71^ one key ethical issue raised by egg-sharing schemes concerns the validity of the consent of the egg sharer, when discounted treatment access is provided. Indeed, a woman may be agreeing to becoming an egg sharer in the first place, only because she does not have the means to pay for her own MAR, and the discount associated with egg sharing becomes the only way for her to afford treatment. In other words, it is moot ‘whether the offer of a free or partially free cycle would jeopardize the voluntariness of the donation’.^72^ This may also turn into a source of discrimination, since only wealthy people can afford to not undergo egg sharing, as they can pay the full price of MAR, whereas poorer aspiring parents may have to agree to egg sharing to try and have a child. This risk is particularly high in the Swiss context due to its peculiarities. Indeed, in Switzerland, MAR is completely unsubsidized, meaning that patients always have to pay for basically the whole treatment. Moreover, treatment in Switzerland is quite expensive: the price of a single in-vitro-fertilisation cycle can be around 8,000–10,000 Swiss Francs (9,400–11,700 USD), thus making cost a real barrier to access (especially if multiple cycles are needed). For these reasons, if the possibility of a substantial discount is offered in exchange for becoming an egg sharer, many people may be highly motivated to become egg sharers for financial reasons. Only those who are financially better off may have a real choice whether they want to share their eggs or not.

Empirical research may be used to dispel some of the fears that egg sharing entails. Indeed, a systematic review^73^ on egg sharing in the UK has shown that there are many reasons why patients choose to become sharers (e.g. for altruistic purposes) other than financial incentives. For example, Gürtin and colleagues^74^ analysed a sample of 48 egg sharers and they conclude in their discussion that:

For some donors, egg-sharing offered a practical option, enabling them to address their financial concerns while helping another. For others, the decision to egg-share was made independently of their financial situation, informed to a greater extent by broader attitudes towards donation and reciprocity.

At the same time, the results of this study say that to the specific question ‘Would you have considered donating some of your eggs if there was no benefit-in-kind (i.e. cheaper IVF treatment) offered?’, only 38.3% of them replied yes, with 19.1% stating no, and 43.6% saying that they were not sure. Moreover, when the authors of the study asked to select from a number of motivations (more than one answer possible) what convinced the participants to take part in egg sharing, 48.9% picked ‘I could not afford my IVF treatment otherwise’ as an answer. This indicates that there is a percentage of people who become egg sharers in exchange for the discounted treatment but would not otherwise do so (e.g. if MAR was covered by basic health insurance). More generally, although studies concerning research on egg sharing are available,^75^ many open questions surrounding this practice remain. In reviewing such literature, Hodson notes that existing empirical literature is limited by ‘self-selection, social desirability bias and financial conflict of interest’.^76^

A 2006 project from Belgium,^77^ relying on retrospective analysis of administrative data, showed that the number of egg sharers dropped by 70% once MAR was fully subsidised by the government (whereas beforehand, many people had to pay out of pocket, and thus turned to egg sharing to get a discount). These numbers are indicative, and they may vary in another context. Yet, the fact remains that in a context where MAR is paid out of pocket, having a discount in exchange for egg sharing may be a significant motivating factor for women to agree to become egg sharers. Indeed, as the authors of the Belgian study put it:

The data show that the discounted treatment is an important motive for more than two-thirds of the women who share their eggs. Although it is difficult to estimate the degree of coercion experienced by these women, it is clear that some of these women would never donate without the cost reduction. Moreover, the system also raises a matter of justice since only women who cannot afford treatment will have to share. The solution however does not lie in the prohibition of compensated egg sharing but in more extended funding of IVF for the financially needy. Egg sharing or partial donation is a perfectly acceptable procedure if integrated in a context in which IVF is funded with public money.^78^

Similar concerns emerged in another study on egg sharing,^79^ where authors noted that ‘the local increase in NHS-funded IVF caused a decline in the number of volunteers for [. . .] egg sharing’, which implies that ‘funding does play a role in the decision to donate eggs’.^80^ The problems that egg sharing raises should not be used as reasons to forbid egg sharing altogether – as we discuss in the next chapter – or to consider any form of discount as compromising the ‘freely given’ nature of the consent. Indeed, empirical research^81^ shows that the decision to become an egg sharer is often motivated by financial constraints, but also by altruistic motives. It should, however, motivate lawmakers to accurately consider the concrete implications that legalising egg donation may have in the Swiss context in terms of egg-sharing practices, in particular regarding socio-economic discrimination. In fact, as long as MAR remains unsubsidized and not covered by basic health insurance, the provision of discounted treatment in exchange for egg sharing is very likely to have a relevant influence on egg sharers’ consent, given the high costs that women can save only if they share oocytes, and on the risk to enhance inequality, as financial compensations will impact more those with less financial means.

## Propositions for addressing the two legal issues

The debate on the egg donation in Switzerland has focused so much on the reasons why the absolute ban should be eliminated that relatively little attention has been given to the various legal questions arising from the legalisation. In the previous sections, we problematised two issues that received little attention, but actually require an in-depth discussion in order to implement the legalisation of egg donation in the best possible way. To contribute to this discussion, we now sketch potential ways to address the legal issues we raised.

With regards to the issue of age limitations for the recipients of egg donation, our proposition is to consider the addition of explicit chronological age limits also in the Swiss context. The main advantages of having explicit chronological age limits are that chronological age is very easy to ascertain, that clear limits enhance transparency towards patients and clinicians, and that uniform limits promote equality in how different fertility centres may treat their patients. A recent survey on this matter conducted in clinics in the US – where no explicit chronological age limits exist – showed that clinics set their own institutional age limits, which turns out to be quite problematic.

This inconsistency [i.e. the fact that – without legal chronological age limits – each clinic decides their own institutional age limits] can create confusion for patients, as it is possible for patients to be eligible for treatment at one center and barred from the same treatment at another. This inconsistency also puts pressure on providers to offer fertility care in situations about which they may have significant concerns. For example, if a patient can be seen at one fertility clinic with no age cut-off and they then go to another clinic expecting similar treatment, the fertility provider is likely to feel pressure to provide that care in order to placate the patient or prevent receiving a bad review.^82^

In fact, a great majority of the clinicians surveyed in the same study indicated that they would like to have clear centralised regulations on age limits. Another problem of not having clear legal guidelines regarding age in MAR was shown by another study conducted in the US. Alberta and colleagues^83^ investigated to what extent agencies adhere to the guideline recommendation (rather than strict legal rule) to recruit women aged above 21 in their advertisements to find egg donors. Authors found out that a great number of agencies disregarded the recommendation and advertised also for younger egg donors. They then correctly remark that

Although the focus of this analysis is compliance with the oocyte donor age guidelines, the results raise broader questions about the efficacy of self-regulation in the industry. The donor age guidelines are presumably among the easiest of ASRM [American Society for Reproductive Medicine]’s self-regulatory guidelines for clinics and agencies to monitor and follow. Yet advertising for donors under the age of 21 appears widespread, suggesting that noncompliance with the preferred use of donors age 21 and older may also be widespread.

In short, given the many interests at play in the sector of MAR, the reliance on self-regulation concerning matters such as age limits is problematic, and legal rules with explicit age limits seem to be the preferable solution.

Naturally, the question remains as to *how* the legislator should design rules containing an explicit chronological age limit. In another publication, we argued that a specific type of chronological age limit for aspiring parents who want to access MAR would be particularly appropriate.^84^ We called these ‘soft’ chronological age limits and explained that they would have to possess three features. First, they should entail the indication of a specific chronological age (i.e. a number) in the law directly. This would provide a useful yardstick for clinicians, thus avoiding that individual clinics or individual healthcare professionals set their own arbitrary limits. It would also help to keep the question of parenting at an advanced age under the public eye, and thus up for scrutiny, and it would help to spread the public health message that fertility has limits. Second, they would need to be exceptionally derogable, meaning that in exceptional cases, professionals should be able to deviate from the age limit, if the right justification is provided. Third, they would have to entail sunset clauses, that is, the obligation for the lawmaker to regularly (e.g. every 5 or 10 years) review them and thus adapt them to socio-technical evolution.

Would this solution – a ‘soft’ chronological age limit – be implementable in the Swiss legal framework? As we outlined in the section ‘The question about a chronological age limit’, some legal commentators and the NEC have opposed the idea of setting explicit chronological age limits in the law. However, at the same time, when the NEC reflected on the issue of age limits for the use of own frozen eggs, they opened up the possibility that a chronological age limit may be justified, if this is done by the legislator directly. Indeed, in their report, they wrote that ‘[t]he question of a fixed age limit on grounds of social ethics is one which is to be answered – if at all – by democratically legitimated legislators’.^85^ It seems therefore that the Swiss legal framework is open to this solution and that – ultimately – it will be only a political decision. In this respect, we support that there is a discussion about a ‘soft’ chronological age limit within the parliament and that an age cap of this kind is considered. In terms of deciding which exact age (number) to set, we recommend to follow the framework by Piek and colleagues.^86^ They suggest to treat chronological age as an intermediate variable (i.e. a proxy) that regulation may set as a criterion not because it is important in itself, but because it helps to approximate a ‘target variable’ – for example, the chances of performing MAR in a safe-enough manner for all parties involved. Following this reasoning, they recommend that regulators first select a suitable target variable and then set an age limit that strongly correlates with this target variable. One possible target variable is the chance of obtaining a live birth. The American Society for Reproductive Medicine indeed stipulated that an MAR treatment where the chances to achieve a live birth are below 1% can be deemed futile, and clinicians should in principle refuse to treat these patients.^87^ Thus, legislators could pick a value in terms of chance of achieving a live birth, and – if enough evidence exists that a specific parental age correlates strongly with chances of a live birth lower than such value – they could pick such specific age as a ‘soft’ chronological age limit. A similar reasoning was applied in setting age limits for the Ontario Fertility Program, a policy which funded MAR in a Canadian Province: Policymakers decided to limit public funding for an IVF cycle to aspiring mothers younger than 43.^88^ This number was chosen ‘based on a recommendation that cumulative live birth rate should be at least 10% to qualify’ and that available evidence at the time showed that from 43 years of age for the aspiring mother had a strong correlation with a cumulative live-birth rate lower than 10%. This is not to say that 43 should be the number chosen for Switzerland (or that the choice of a 10% mark is appropriate), but to show how a transparent and accountable process for setting a suitable ‘soft’ chronological age limit in Switzerland could proceed.

With regards to the issue of regulating egg procurement, we first want to highlight that it is regrettable how this has received relatively very little attention. One reason for this may be that questioning where the eggs would come from has often been used as a political argument by members of parliament to either postpone or oppose the legalisation of egg donation.^89^ As a consequence, policymakers who pushed for egg donation to become lawful may have sidestepped the issue by claiming that it is appropriate to discuss it only after the legalisation is approved *in principle*.^90^ However, this has led to a situation where the delicate aspect of how to regulate the procurement of eggs is not discussed in depth. As we briefly outlined, only one small survey has investigated Swiss women’s willingness to donate eggs and explored what would motivate them.^91^ Among the different ways how egg procurement could be regulated, the solution of facilitating egg sharing is often mentioned. And yet, we have shown that in the context of Switzerland – where MAR is not covered by basic health insurance and is very expensive – egg-sharing schemes may easily lead to discriminatory treatment and undue inducement, especially if coupled with discounted treatment for the egg sharer. As we hinted at the end of the section ‘Regulating egg procurement and the delicate practice of egg sharing’, this does not mean that egg sharing should be banned altogether. Indeed, we agree with Scott that the consent by egg sharers cannot be considered universally invalid only because a financial compensation is included, and in the absence of extensive public funding, egg sharing may be the only way for some aspiring parents to access MAR.^92^ Thus, the most equitable solution to avoid that the legalisation of egg sharing creates a strong undue inducement for poorer aspiring parents to share their eggs only to pay for their own reproductive treatment is to provide at least basic public funding for MAR. If some public funding or coverage through basic social health insurance were set up, the risk that people would agree to become egg sharers just because of the discount to their own treatment would be reduced.^93^ Some discounts for those who provide eggs may still be permitted, but they should be tightly regulated, so that sharing eggs does not become the ‘only’ option for those who struggle to afford MAR. More importantly, like Scott underlines in her conclusions,^94^ legalising egg sharing should not be used by the government as a way to delegate MAR financing to the market and avoid providing support through public money. On the contrary, the discussion around the legalisation of egg donation and egg sharing shows even more clearly how important it is to promote some basic public funding of MAR in the near future. This would be particularly important for another reason connected to the issue of parental age which we tackled above. Indeed, as we have learned while conducting our interviews with healthcare professionals working in MAR, the absence of any substantial coverage through basic social health insurance generates another paradoxical situation: People may have to wait years until they can eventually afford MAR, while, at the same time, waiting impacts negatively on their fertility and reduces the chances that MAR will be successful.

Another potential alternative to facilitate egg procurement in the Swiss context is to promote the donation of unused eggs that had originally been collected as part of elective egg freezing, that is, when women decide to freeze their eggs for non-medical reasons. This practice is highly marketed in Switzerland, although it is unknown how widely used it actually is, especially considering its high costs.^95^ If eggs collected in this context remain unused, they could constitute another potential source for egg donation – as has been suggested elsewhere.^96^ However, this pathway entails a series of serious ethico-legal and financial questions.^97^ Moreover, it may require a further adaptation of some Swiss legal rules, which nowadays permit social egg freezing^98^ but mandate, for example, that eggs can be preserved only for 10 years. Finally, there would be the need for the law to require those who decide to share part of their unused frozen eggs with other couples to undergo specific counselling given that choosing what to do with frozen oocytes involves a particularly complex decision-making process.^99^ A protocol on how to conduct such counselling in a standardised and ethically-informed way has recently been proposed.^100^

## Concluding remarks

In this article, we examined the special case of Switzerland, which remains one of the very few countries in Europe still banning egg donation in its entirety. We explored the political reasons why an outright ban was implemented in the first place and how it is now being removed. Thereafter, we scrutinised two crucial issues concerning *how* egg donation will be legalised, rather than *whether* it should be legalised. We discussed the challenges posed by the legalisation of egg donation in terms of chronological age limits for access to MAR, in a country like Switzerland that – so far – has refused to put any specific age limits in the field of reproductive medicine. We then showed the open questions that the legalisation of egg donation will raise in respect to the procurement of eggs, and we examined some problems that may arise from the legalisation of egg-sharing schemes as a way to find eggs, especially in the Swiss context where MAR is currently unsubsidized and thus patients need to pay for it out of pocket. Finally, we briefly outlined two potential solutions for addressing the issues that we raised, namely that of introducing soft chronological age limits and that of providing some basic public funding for MAR, if egg sharing is introduced by the regulation as a way to procure eggs.

In conclusion, we stress once more that it cannot be ignored how the legalisation of egg donation in Switzerland brings with itself a series of questions about the concrete implications that it will have on MAR practice. These should not be used as arguments to hamper the legalisation of egg donation, but – on the contrary – to ensure that the legal conditions under which it will be permitted provide the best possible guarantee of the rights of aspiring parents and their children.

## Footnotes

1. Patrick Präg and Melinda C. Mills, ‘Assisted Reproductive Technology in Europe: Usage and Regulation in the Context of Cross-Border Reproductive Care’, in Michaela Kreyenfeld and Dirk Konietzka, eds, *Childlessness in Europe: Contexts, Causes, and Consequences*, Demographic Research Monographs (Cham: Springer International Publishing, 2017), pp. 289–309. DOI: 10.1007/978-3-319-44667-7_14.

2. K. Berg Brigham, B. Cadier, and K. Chevreul, ‘The Diversity of Regulation and Public Financing of IVF in Europe and Its Impact on Utilization’, *Human Reproduction* 28(3) (March 1, 2013): 666–675. DOI: 10.1093/humrep/des418; C. Calhaz-Jorge, C. De Geyter, M. S. Kupka, C. Wyns, E. Mocanu, T. Motrenko, G. Scaravelli, J. Smeenk, S. Vidakovic, V. Goossens, ‘Survey on ART and IUI: Legislation, Regulation, Funding and Registries in European Countries,’ *Human Reproduction Open* 2020, no. 1 (January 1, 2020): hoz044. DOI: 10.1093/hropen/hoz044.

3. Audrey Lebret, ‘The European Court of Human Rights and the Framing of Reproductive Rights’, *Droits Fondamentaux* 18 (2020).

4. Wannes Van Hoof and Guido Pennings, ‘Extraterritorial Laws for Cross-Border Reproductive Care: The Issue of Legal Diversity’, *European Journal of Health Law* 19(2) (April 2012): 187–200. DOI: 10.1163/157180912x628226.

5. Fertility Europe, European Atlas of Fertility Treatment Policies available at https://fertilityeurope.eu/european-atlas-of-fertility-treatment-policies/ (accessed 7 June 2021).

6. S.H. AND OTHERS v. AUSTRIA, No. Application no. 57813/00 (European Court of Human Rights - Grand Chamber 2011). The main matter of contention focused mainly on whether the ban on egg donation was compliant with article 8 of the European Convention of Human Rights.

7. Republik Österreich, ’Bundesgesetz, Mit Dem Regelungen Über Die Medizinisch Unterstützte Fortpflanzung Getroffen Werden (Fortpflanzungsmedizingesetz – FMedG)’, Pub. L. No. BGBl. Nr. 275/1992 (n.d.), available at https://www.ris.bka.gv.at/GeltendeFassung.wxe?Abfrage=Bundesnormen&Gesetzesnummer=10003046.

8. Italian Constitutional Court, JUDGMENT NO. 162 YEAR 2014 (Italian Constitutional Court 2014).

9. The only other European countries which do not allow egg donation are Bosnia & Herzegovina, Germany, Luxembourg, and Turkey. See The European IVF-Monitoring Consortium (EIM) for the European Society of Human Reproduction and Embryology (ESHRE) et al., ‘Survey on ART and IUI: Legislation, Regulation, Funding, and Registries in European Countries—an Update’, *Human Reproduction* 39(9) (1 September 2024): 1909–1924, DOI: 10.1093/humrep/deae163.

10. ‘Federal Act on Medically Assisted Reproduction - Reproductive Medicine Act (RMA)’, 18 December 1998, available at https://www.fedlex.admin.ch/eli/cc/2000/554/en. More details on this piece of legislation are provided below.

11. Schweizerische Akademie der Medizinischen Wissenschaften, ‘Medizinisch-Ethische Richtlinien Für Die Ärztlich Assistierte Fortpflanzung’, 1990, available at https://www.samw.ch/dam/jcr:ef687f57-0731-4a5c-aaf3-29bf4bbc6cd4/richtlinien_samw_aerztlich_assistierte_fortpflanzung_1990.pdf.

12. For a more detailed story of this period, see Bernhard Rütsche and Ladina Zegg, ‘Allgemeiner Teil / III. Teil: Quellen Und Entwicklungsgeschichte Des Fortpflanzungsmedizinrechts / I. - III. Fortpflanzungsmedizingesetz (FMedG)’, Andrea Büchler and Bernhard Rütsche eds, in *Fortpflanzungsmedizingesetz (FMedG)*, SHK - Stämflis Handkommentar (Bern: Stämpfli Verlag AG, 2020), pp. 45–65.

13. The whole history of the different steps of the initiatives can be consulted online, see Eidgenössische Volksinitiative ‘Gegen Missbräuche Der Fortpflanzungs- Und Gentechnologie Beim Menschen’, available at https://www.bk.admin.ch/ch/d/pore/vi/vis182.html (accessed 24 August 2023).

14. Counter proposals are alternative changes to the constitution that the government and the parliament propose as a reply to the submission of a popular initiative. They normally try to strike a balance between the changes proposed by the popular initiative and the vision of the government and other elected representatives. Such counter proposals are then placed on the ballot together with the proposals contained in the popular initiative, and citizens then express their preferences.

15. ‘Federal Act on Medically Assisted Reproduction’, 18 December 1998 available at https://www.fedlex.admin.ch/eli/cc/2000/554/en.

16. The chamber of the parliament where two elected councillors of each cantons (Federal states) are represented, up to a total of 46 members. It defends the interests of the cantons themselves. It is roughly corresponding to the Senate in the United States of America.

17. The chamber of the parliament which directly represents Swiss people. It is composed of 200 members, elected based on electoral colleges influenced by the amount of population in a certain area.

18. Bernhard Rütsche and Ladina Zegg, ‘Allgemeiner Teil / III. Teil: Quellen Und Entwicklungsgeschichte Des Fortpflanzungsmedizinrechts / IV. - VI. Fortpflanzungsmedizingesetz (FMedG)’, in Andrea Büchler and Bernhard Rütsche eds, *Fortpflanzungsmedizingesetz (FMedG)*, SHK - Stämflis Handkommentar (Bern: Stämpfli Verlag AG, 2020), pp. 65–97.

19. Schweizer Bundesrat, ‘Entwurf Und Botschaft Vom 26. Juni 1996 Über Die Initiative Für Menschenwürdige Fortpflanzung Und Zum Fortpflanzungsmedizingesetz’, 1996, https://www.bj.admin.ch/dam/bj/de/data/gesellschaft/gesetzgebung/archiv/fortpflanzungsmedizin/bot-fortpflanzungsmedizin-d.pdf.download.pdf/bot-fortpflanzungsmedizin-d.pdf.

20. Bundesrat. Our translation.

21. Andrea Büchler and Sandro Clausen, ‘Art. 4 Verbotene Praktiken’, in Andrea Büchler and Bernhard Rütsche eds, *Fortpflanzungsmedizingesetz (FMedG)*, SHK - Stämflis Handkommentar (Bern: Stämpfli Verlag AG, 2020), pp. 236–248.

22. Andrea Büchler, ‘Die Eizellenspende in Der Schweiz de Lege Lata Und de Lege Ferenda’, Rechtsgutachten verfasst im Auftrag des Bundesamtes für Gesundheit, January 2013 available at https://www.ius.uzh.ch/dam/jcr:a089bc47-2535-40c6-8931-ebba03ddd7b4/Buechler_RechtsgutachtenEizellenspende.pdf.

23. Büchler and Clausen, ‘Art. 4 Verbotene Praktiken’.

24. Eva Maria Belser and Alexandra Jungo, ‘Elternschaft Im Zeitalter Medizinischer Machbarkeit. Das Recht Auf Achtung Des Kinderwunsches Und Seine Schranken’, *Zeitschrift Für Schweizerisches Recht* 135(3) (2016).

25. NEK - Nationale Ethikkommission, ‘Die Medizinisch Unterstützte Fortpflanzung. Ethische Überlegungen Und Vorschläge Für Die Zukunft’, 2013 available at https://www.nek-cne.admin.ch/inhalte/Themen/Stellungnahmen/NEK_Fortpflanzungsmedizin_De.pdf.

26. For a summary, see Büchler and Clausen, ‘Art. 4 Verbotene Praktiken’.

27. Swiss Federal Parliament, ‘Kinderwunsch Erfüllen, Eizellenspende Für Ehepaare Legalisieren - Motion 21.4341’, 2021, available at https://www.parlament.ch/de/ratsbetrieb/suche-curia-vista/geschaeft?AffairId=20214341.

28. Swiss National Council, ‘Motion WBK-N. Kinderwunsch Erfüllen, Eizellenspende Für Ehepaare Legalisieren’, 17 March 2022 available at https://www.parlament.ch/de/ratsbetrieb/amtliches-bulletin/amtliches-bulletin-die-verhandlungen?SubjectId=56619.

29. Swiss Council of States, Motion WBK-N. Kinderwunsch Erfüllen, Eizellenspende Für Ehepaare Legalisieren available at https://www.parlament.ch/de/ratsbetrieb/amtliches-bulletin/amtliches-bulletin-die-verhandlungen?SubjectId=57928 (accessed 24 August 2023).

30. Absolute certainty can never be guaranteed in the Swiss law-making system even in cases like this where both chambers have voted in favour of a change in legislation. The reason for that is that – even if approved by the parliament – new laws or changes to existing law can be challenged to a popular referendum, as long as 50,000 signatures are collected within 100 days of their publication. However, given the political situation, in this case, chances are quite high.

31. Family Building at Advanced Parental Age: An Interdisciplinary Approach, n.d., available at https://data.snf.ch/grants/grant/197415 (last accessed 9 September 2024).

32. Andrea Büchler and Karène Parizer, ‘Maternal Age in the Regulation of Reproductive Medicine – A Comparative Study’, *International Journal of Law, Policy and the Family* 31(3) (1 December 2017): 269–290, DOI: 10.1093/lawfam/ebx007; Berg Brigham, Cadier, and Chevreul, ‘The Diversity of Regulation and Public Financing of IVF in Europe and Its Impact on Utilization’.

33. An example of a non-chronological age limit is the Italian law on medically assisted reproduction, which determines that access can be granted only to ‘adult couples [. . .] in potentially fertile age’. Legge 19 febbraio 2004, n. 40 - Norme in materia di procreazione medicalmente assistita, available at https://www.trovanorme.salute.gov.it/norme/dettaglioAtto?id=4538&completo=true (accessed 21 May 2024). Original in Italian, our translation.

34. This article is interpreted in the sense that the couple ‘as a whole’ has to be able to ensure that. Of note, access to single parents is not allowed in Switzerland.

35. This will always be the father, for the reasons explained further on in the article.

36. Andrea Büchler and Sandro Clausen, ‘Art. 3 Kindeswohl /IV. - VII.’, in Andrea Büchler and Bernhard Rütsche eds, *Fortpflanzungsmedizingesetz (FMedG)*, SHK - Stämflis Handkommentar (Bern: Stämpfli Verlag AG, 2020), pp. 212–235.

37. Andrea Martani, Eva De Clercq, Christian De Geyter, Guido Pennings, Tenzin Wangmo and Bernice S. Elger, ‘Deconstructing Age(s): An Analysis of the Different Conceptions of Age as a Legal Criterion for Access to Assisted Reproductive Technologies’, *Journal of Law and the Biosciences* 9(2) (1 July 2022): lsac036. DOI: 10.1093/jlb/lsac036.

38. Unless women have frozen their eggs, a practice which is not explicitly regulated in Switzerland, but considered legal. See NEK - Nationale Ethikkommission, ‘Social Egg Freezing - Ethical Reflections’, 2017, Social egg freezing - ethical reflections.

39. Kinderwunschzentrum - Universitätsspital Zürich, Die Häufigsten Fragen Rund Um Die Kinderwunschbehandlung available at https://www.usz.ch/kinderwunsch/die-haeufigsten-fragen-rund-um-die-kinderwunschbehandlung/ (accessed 24 August 2023). Our translation, original in German.

40. NEK - Nationale Ethikkommission, ‘Die Eizellenspende - Ethische Und Rechtliche Erwägungen’, 2022, available at https://www.nek-cne.admin.ch/inhalte/Themen/Stellungnahmen/de/NEK-CNE_Stellungnahme_Eizellenspende_DE.pdf.

41. A different situation is the one where own frozen eggs are used, that were extracted and frozen at an earlier age. This is possible in Switzerland, and the NEC recognized that introducing a specific chronological age limit for the use of frozen eggs may be acceptable, as long as it is established ‘by democratically legitimated legislators’. See NEK - Nationale Ethikkommission, ‘Social Egg Freezing - Ethical Reflections’.

42. Richard J. Paulson, Robert Boostanfar, Peyman Saadat, Eliran Mor, David E. Tourgeman, Cristin C. Slater, Mary M. Francis, and John K. Jain, ‘Pregnancy in the Sixth Decade of Life: Obstetric Outcomes in Women of Advanced Reproductive Age’, *JAMA* 288(18) (13 November 2002): 2320. DOI: 10.1001/jama.288.18.2320.; M. V. Sauer, R. J. Paulson, and R. A. Lobo, ‘Pregnancy: Oocyte Donation to Women of Advanced Reproductive Age: Pregnancy Results and Obstetrical Outcomes in Patients 45 Years and Older’, *Human Reproduction* 11(11) (1 November 1996): 2540–2543, DOI: 10.1093/oxfordjournals.humrep.a019155.

43. Nolwenn Bühler, Egg Donation and IVF with Donated Eggs: Lessons to Be Learned from Other Countries. Social Science Report on Behalf of the Federal Office of Public Health (Bern, 2014) available at https://www.aramis.admin.ch/Dokument.aspx?DocumentID=3545.

44. Barbara Achermann, Andrea Büchler, and Laura Perler, ‘Sie Wollen Die Eizellspende Legalisieren, Ohne Die Details Zu Klären’ – ‘Der Kinderwunsch Ist Verfassungsrechtlich Geschützt’ (Interview)’, *DIE ZEIT*, 12 May 2022. DOI: 10.5167/UZH-218757.

45. Anna Nüssli, Gideon Sartorius, Isabelle Bienz, Rebecca Moffat, and Christian De Geyter, ‘Rahmenbedingungen für die Bereitschaft junger Schweizer Frauen zur Spende von Eizellen’, *Schweizerische Ärztezeitung* 95(7) (11 February 2014). DOI: 10.4414/saez.2014.02142.

46. See, e.g. Timothy Bracewell-Milnes, James C. Holland, Benjamin P. Jones, Srdjan Saso, Paula Almeida, Kate Maclaran, Julian Norman-Taylor, Dimitrios Nikolaou, Nishel M. Shah, Mark Johnson, and Meen-Yau Thum, ‘Exploring the Knowledge and Attitudes of Women of Reproductive Age from the General Public towards Egg Donation and Egg Sharing: A UK-Based Study’, *Human Reproduction* 36(8) (19 July 2021): 2189–2201. DOI: 10.1093/humrep/deab157.

47. The Ethics Committee of the American Society for Reproductive Medicine, ‘Financial Compensation of Oocyte Donors: An Ethics Committee Opinion’, *Fertility and Sterility* 116(2) (August 2021): 319–325, DOI: 10.1016/j.fertnstert.2021.03.040.

48. G. Pennings, G. de Wert, F. Shenfield, J. Cohen, B. Tarlatzis, and P. Devroey, ‘ESHRE Task Force on Ethics and Law 12: Oocyte Donation for Non-Reproductive Purposes’, *Human Reproduction* 22(5) (1 May 2007): 1210–1213, DOI: 10.1093/humrep/dem025.

49. Eshre Task Force On Ethics And Law, ‘III. Gamete and Embryo Donation’, *Human Reproduction* 17(5) (1 May 2002): 1407–1408. DOI: 10.1093/humrep/17.5.1407.

50. Guido Pennings, ‘Central Role of Altruism in the Recruitment of Gamete Donors’, *Monash Bioethics Review* 33(1) (March 2015): 78–88. DOI: 10.1007/s40592-015-0019-x.

51. Sonja Goedeke, Daniel Shepherd, and Iolanda S Rodino, ‘Support for Recognition and Payment Options for Egg and Sperm Donation in New Zealand and Australia’, *Human Reproduction* 35(1) (1 January 2020): 117–129, DOI: 10.1093/humrep/dez257. In the introduction of this study, readers can also find a general overview of the arguments in favor and against compensations for egg donation.

52. Art 21 of the ‘Federal Act on Medically Assisted Reproduction - Reproductive Medicine Act (RMA)’.

53. NEK - Nationale Ethikkommission, ‘Die Eizellenspende - Ethische Und Rechtliche Erwägungen’.

54. Achermann, Büchler, and Perler, ‘Sie Wollen Die Eizellspende Legalisieren, Ohne Die Details Zu Klären’ – ‘Der Kinderwunsch Ist Verfassungsrechtlich Geschützt’ (Interview)’.

55. Donors only receive an indemnity, which, however, is quite substantial. For example, in a sperm bank based in the Basel area, donors receive 150 Swiss Francs (ca. 170 US dollars) per appointment up to a maximum of 1,500 Swiss Francs (ca. 1,700 US dollars) – see fertisuisse, Familien Zum Glück Verhelfen. Werden Sie Samenspender available at https://www.fertisuisse-samenspende.ch/ (accessed 31 August 2023). In a sperm bank in the area of Zürich, an indemnity of 2,000 Swiss Francs (ca. 2,265 US dollars) per donation is paid – see OVA IVF Clinic Zurich, ‘Spesenentschädigung’ available at https://donors.ch/ (accessed 31 August 2023).

56. The actual number of sperm donors is difficult to determine. According to the official Sperm Donor Registry, there were only 61 new sperm donors between 2019 and 2022 – see Federal Office for Civil Registration & Federal Office of Public Health, Sperm Donor-Conceived Children available at https://www.bag.admin.ch/bag/en/home/zahlen-und-statistiken/zahlen-fakten-zu-fortpflanzungsmedizin/kinder-aus-samenspende.html (accessed 31 August 2023). However, there is an obligation to register only the sperm donors whose gametes have then been used to give birth to a new child, and not the total number of people who have donated – who is thus surely higher than 61.

57. Bühler, ‘Egg Donation and IVF with Donated Eggs: Lessons to Be Learned from Other Countries. Social Science Report on Behalf of the Federal Office of Public Health’.

58. Y. Englert, ‘Oocyte Donation: Ethics of Oocyte Donation Are Challenged by the Health Care System’, *Human Reproduction* 11(11) (1 November 1996): 2353–2354. DOI: 10.1093/oxfordjournals.humrep.a019113; P. Baetens, ‘Counselling Couples and Donors for Oocyte Donation: The Decision to Use Either Known or Anonymous Oocytes’, *Human Reproduction* 15(2) (1 February 2000): 476–484. DOI: 10.1093/humrep/15.2.476.

59. NEK - Nationale Ethikkommission, ‘Die Eizellenspende - Ethische Und Rechtliche Erwägungen’; Büchler, ‘Die Eizellenspende in Der Schweiz de Lege Lata Und de Lege Ferenda’.

60. Roland Fankhauser, ‘Art. 22 Verwendung Gespendeter Samenzellen’, in Andrea Büchler and Bernhard Rütsche eds, *Fortpflanzungsmedizingesetz (FMedG)*, SHK - Stämflis Handkommentar (Bern: Stämpfli Verlag AG, 2020), pp. 571–576.

61. This term is often used misleadingly in Swiss debates as a synecdoche to refer to both egg-sharing and freeze and share programs. For example, see UZH News, ‘Should Egg Donation Be Legalized in Switzerland?’ 21 December 2022, available at https://www.news.uzh.ch/en/articles/news/2022/Should-Egg-Donation-Be-Legalized-in-Switzerland.html. Freeze and share practices are those where a woman who extracts and freezes her own eggs for social or medical reasons then decides to share part of them with another person. This practice may also be described as a donation and will likely become lawful once egg donation is legalized in Switzerland, and in our study, one participant we interviewed also expressed her frustration that she cannot donate her unused frozen eggs. Nevertheless, it should be distinguished from egg-sharing practices in the strict sense, as it is explained below in the text.

62. SRF News, ‘Ethiker Befürworten Eizellenspende’, 19 January 2014, available at https://www.srf.ch/news/schweiz/schweiz-ethiker-befuerworten-eizellenspende; ‘Der Kindermacher’, *Tagesanzeiger*, 22 August 2020, available at https://www.tagesanzeiger.ch/der-kindermacher-773916815503.

63. Ibid., at Reference 59.

64. Human Fertilisation & Embryology Authority, Donating Your Eggs available at https://www.hfea.gov.uk/donation/donors/donating-your-eggs/ (accessed 24 August 2023).

65. Rune Klingenberg, ‘Freeze-and-share: New schemes, old ethics?’ *Bioethics Update* 7(1) (4 June 2021): 5995, DOI: 10.24875/BUP.M20000003; Alex Polyakov and Genia Rozen, ‘Social Egg Freezing and Donation: Waste Not, Want Not’, *Journal of Medical Ethics* 47(12) (December 2021): e73–e73. DOI: 10.1136/medethics-2020-106607.

66. E. Blyth, ‘Subsidized IVF: The Development of “egg Sharing” in the UK’, *Human Reproduction* 17(12) (1 December 2002): 3254–3259.DOI: 10.1093/humrep/17.12.3254.

67. Eric G Simons and Kamal K Ahuja, ‘Egg-Sharing: An Evidence Based Solution to Donor Egg Shortages’, *The Obstetrician & Gynaecologist* 7(2) (April 2005): 112–116. DOI: 10.1576/toag.7.2.112.27069.

68. Büchler, ‘Die Eizellenspende in Der Schweiz de Lege Lata Und de Lege Ferenda’; NEK - Nationale Ethikkommission, ‘Die Eizellenspende - Ethische Und Rechtliche Erwägungen’.

69. Our translation. Original in German ‘Nach Ansicht der NEK gibt es auch keine überzeugenden Gründe, das egg sharing zu untersagen, d. h. die Möglichkeit, Eizellen zu spenden, die im Rahmen einer IVF gewonnen wurden und nicht mehr benötigt werden. Beim egg sharing ist besonders darauf zu achten, dass die IVF-Behandlung der Spenderin nicht durch die Spende beeinträchtigt wird’. See NEK - Nationale Ethikkommission, ‘Die Eizellenspende - Ethische Und Rechtliche Erwägungen’.

70. Büchler, ‘Die Eizellenspende in Der Schweiz de Lege Lata Und de Lege Ferenda’; NEK - Nationale Ethikkommission, ‘Die Eizellenspende - Ethische Und Rechtliche Erwägungen’.

71. Eric Blyth and Berenice Golding, ‘Egg Sharing: A Practical and Ethical Option in IVF?’ *Expert Review of Obstetrics & Gynecology* 3(4) (July 2008): 465–473. DOI: 10.1586/17474108.3.4.465.

72. G. Pennings, J. de Mouzon, F. Shenfield, A. P. Ferraretti, T. Mardesic, A. Ruiz, and V Goossens, ‘Socio-Demographic and Fertility-Related Characteristics and Motivations of Oocyte Donors in Eleven European Countries’, *Human Reproduction* 29(5) (1 May 2014): 1076–1089. DOI: 10.1093/humrep/deu048.

73. Timothy Bracewell-Milnes et al., ‘A Systematic Review Investigating Psychosocial Aspects of Egg Sharing in the United Kingdom and Their Potential Effects on Egg Donation Numbers’, *Human Fertility* 21(3) (3 July 2018): 163–173, DOI: 10.1080/14647273.2017.1329554.

74. Zeynep B. Gürtin, Kamal K. Ahuja, and Susan Golombok, ‘Egg-Share Donors’ and Recipients’ Knowledge, Motivations and Concerns: Clinical and Policy Implications’, *Clinical Ethics* 7(4) (December 2012): 183–192, DOI: 10.1258/ce.2012.012024.

75. Erica Haimes and Ken Taylor, ‘What Is the Role of Reduced IVF Fees in Persuading Women to Volunteer to Provide Eggs for Research? Insights from IVF Patients Volunteering to a UK ‘Egg Sharing for Research’ Scheme’, *Human Fertility* 16(4) (December 2013): 246–251. DOI: 10.3109/14647273.2013.803164; K. K. Ahuja, B. J. Mostyn, and E. G. Simons, ‘Egg Sharing and Egg Donation: Attitudes of British Egg Donors and Recipients’, *Human Reproduction* 12(12) (1 December 1997): 2845–2852. DOI: 10.1093/humrep/12.12.2845; Gürtin et al., ‘Egg-Share Donors’ and Recipients’ Knowledge, Motivations and Concerns’.

76. Nathan Hodson, ‘Differences between Sperm Sharing and Egg Sharing Are Morally Relevant’, *Journal of Medical Ethics* 49(1) (January 2023): 60–64. DOI: 10.1136/medethics-2021-107887.

77. Guido Pennings and Paul Devroey, ‘Subsidized In-Vitro Fertilization Treatment and the Effect on the Number of Egg Sharers’, *Reproductive BioMedicine Online* 13(1) (January 2006): 8–10. DOI: 10.1016/S1472-6483(10)62009-3.

78. Pennings and Devroey ‘Subsidized In-Vitro Fertilization Treatment and the Effect on the Number of Egg Sharers’.

79. Haimes and Taylor, ‘What Is the Role of Reduced IVF Fees in Persuading Women to Volunteer to Provide Eggs for Research?’

80. Anna Tippett, ‘Reproductive Rights Where Conditions Apply: An Analysis of Discriminatory Practice in Funding Criteria against Would-Be Parents Seeking Funded Fertility Treatment in England’, *Human Fertility* 26(3) (27 May 2023): 483–493, DOI: 10.1080/14647273.2022.2164746.

81. Like the review mentioned above, i.e. Bracewell-Milnes et al., ‘A Systematic Review Investigating Psychosocial Aspects of Egg Sharing in the United Kingdom and Their Potential Effects on Egg Donation Numbers’.

82. Jessica H. Selter, Julia Woodward, and Shelby Neal, ‘Survey Assessing Policies Regarding Patient Age and Provision of Fertility Treatment in the United States’, *Journal of Assisted Reproduction and Genetics* 40(9) (September 2023): 2117–2127. DOI: 10.1007/s10815-023-02877-x.

83. Hillary B. Alberta, Roberta M. Berry, and Aaron D. Levine, ‘Compliance with Donor Age Recommendations in Oocyte Donor Recruitment Advertisements in the USA’, *Reproductive BioMedicine Online* 26(4) (April 2013): 400–405. DOI: 10.1016/j.rbmo.2012.11.011.

84. Andrea Martani, Eva De Clercq, Christian De Geyter, Guido Pennings, Tenzin Wangmo, and Bernice Simone Elger, ‘Deconstructing Age(s): An Analysis of the Different Conceptions of Age as a Legal Criterion for Access to Assisted Reproductive Technologies’. In this publication, we also reflect on why it is important that the law explicitly mentions chronological age as a criterion for accessing MAR.

85. NEK - Nationale Ethikkommission, ‘Social Egg Freezing - Ethical Reflections’.

86. Steven R. Piek, Guido Pennings, and Veerle Provoost, ‘Age-Based Restrictions on Reproductive Care: Discerning the Arbitrary from the Necessary’, *Theoretical Medicine and Bioethics*, 11 October 2023. DOI: 10.1007/s11017-023-09648-w.

87. Ethics Committee of the American Society for Reproductive Medicine, ‘Fertility Treatment When the Prognosis Is Very Poor or Futile: An Ethics Committee Opinion’, *Fertility and Sterility* 111(4) (April 2019): 659–663. DOI: 10.1016/j.fertnstert.2019.01.033.

88. Arielle Cantor, Lynn Meng, Andrea Lanes, and Ellen M. Greenblatt, ‘The Effect of the Ontario Fertility Program on IVF Utilization and Outcomes in Women of Advanced Reproductive Age’, *Reproductive BioMedicine Online* 46(2) (February 2023): 410–416. DOI: 10.1016/j.rbmo.2022.05.014.

89. This is evident in the two parliamentary debates quoted above, namely Swiss Council of States, ‘Motion WBK-N. Kinderwunsch Erfüllen, Eizellenspende Für Ehepaare Legalisieren’; Swiss National Council, ‘Motion WBK-N. Kinderwunsch Erfüllen, Eizellenspende Für Ehepaare Legalisieren’.

90. Swiss Council of States, ‘Motion WBK-N. Kinderwunsch Erfüllen, Eizellenspende Für Ehepaare Legalisieren’; Swiss National Council, ‘Motion WBK-N. Kinderwunsch Erfüllen, Eizellenspende Für Ehepaare Legalisieren’.

91. Nüssli et al., ‘Rahmenbedingungen für die Bereitschaft junger Schweizer Frauen zur Spende von Eizellen’.

92. Rosamund Scott, ‘Egg-Sharing, Motivation and Consent: Ethical, Legal and Policy Issues’, in Susan Golombok, Rosamund Scott, John B. Appleby, Martin Richards, and Stephen Wilkinson eds, *Regulating Reproductive Donation* 1st ed. (Cambridge University Press, 2015), pp. 259–290, DOI: 10.1017/CBO9781316117446.013.

93. Of course, this may – on the other hand – reduce the number of eggs which are available for egg receivers. Egg scarcity may be a serious problem both from a medical (e.g. longer waiting time) and a human rights perspective (e.g. importing eggs from countries with questionable legal provision on egg retrieval). A thorough discussion of this issue falls outside the scope of this article.

94. Scott, ‘Egg-Sharing, Motivation and Consent’.

95. D. Wunder, ‘Social Freezing in Switzerland and Worldwide - a Blessing for Women Today?’ *Swiss Medical Weekly*, 27 February 2013. DOI: 10.4414/smw.2013.13746.

96. Bruno Imthurn and Katharina Schiessl, ‘«Social Egg Freezing»’, *Swiss Medical Forum - Schweizerisches Medizin-Forum*, 23 October 2019. DOI: 10.4414/smf.2019.08391.

97. Guido Pennings, ‘When Elective Egg Freezers Become Egg Donors: Practical and Ethical Issues’, *Reproductive BioMedicine Online* 47(1) (July 2023): 151–156. DOI: 10.1016/j.rbmo.2023.03.019.

98. NEK - Nationale Ethikkommission, ‘Social Egg Freezing - Ethical Reflections’.

99. Lucy E. Caughey, Katherine M. White, Sarah Lensen, and Michelle Peate, ‘Elective Egg Freezers’ Disposition Decisions: A Qualitative Study’, *Fertility and Sterility* 120(1) (July 2023): 145–160. DOI: 10.1016/j.fertnstert.2023.02.022.

100. Alexis Heng Boon Chin, Jean-Didier Bosenge Nguma, Charles Nkurunziza, Ningyu Sun, and Guoqing Tong, ‘Counseling Elective Egg Freezing Patients Considering Donation of Unused Surplus Frozen Eggs for Fertility Treatment’, *Asian Bioethics Review*, 29 November 2023. DOI: 10.1007/s41649-023-00268-z.

